# Spatial Variation in Nutrient and Water Color Effects on Lake Chlorophyll at Macroscales

**DOI:** 10.1371/journal.pone.0164592

**Published:** 2016-10-13

**Authors:** C. Emi Fergus, Andrew O. Finley, Patricia A. Soranno, Tyler Wagner

**Affiliations:** 1 Department of Fisheries and Wildlife, Michigan State University, East Lansing, Michigan, United States of America; 2 Departments of Forestry and Geography, Michigan State University, East Lansing, Michigan, United States of America; 3 U.S. Geological Survey, Pennsylvania Cooperative Fish & Wildlife Research Unit, Pennsylvania State University, University Park, Pennsylvania, United States of America; University of Hyogo, JAPAN

## Abstract

The nutrient-water color paradigm is a framework to characterize lake trophic status by relating lake primary productivity to both nutrients and water color, the colored component of dissolved organic carbon. Total phosphorus (TP), a limiting nutrient, and water color, a strong light attenuator, influence lake chlorophyll *a* concentrations (CHL). But, these relationships have been shown in previous studies to be highly variable, which may be related to differences in lake and catchment geomorphology, the forms of nutrients and carbon entering the system, and lake community composition. Because many of these factors vary across space it is likely that lake nutrient and water color relationships with CHL exhibit spatial autocorrelation, such that lakes near one another have similar relationships compared to lakes further away. Including this spatial dependency in models may improve CHL predictions and clarify how well the nutrient-water color paradigm applies to lakes distributed across diverse landscape settings. However, few studies have explicitly examined spatial heterogeneity in the effects of TP and water color together on lake CHL. In this study, we examined spatial variation in TP and water color relationships with CHL in over 800 north temperate lakes using spatially-varying coefficient models (SVC), a robust statistical method that applies a Bayesian framework to explore space-varying and scale-dependent relationships. We found that TP and water color relationships were spatially autocorrelated and that allowing for these relationships to vary by individual lakes over space improved the model fit and predictive performance as compared to models that did not vary over space. The magnitudes of TP effects on CHL differed across lakes such that a 1 μg/L increase in TP resulted in increased CHL ranging from 2–24 μg/L across lake locations. Water color was not related to CHL for the majority of lakes, but there were some locations where water color had a positive effect such that a unit increase in water color resulted in a 2 μg/L increase in CHL and other locations where it had a negative effect such that a unit increase in water color resulted in a 2 μg/L decrease in CHL. In addition, the spatial scales that captured variation in TP and water color effects were different for our study lakes. Variation in TP–CHL relationships was observed at intermediate distances (~20 km) compared to variation in water color–CHL relationships that was observed at regional distances (~200 km). These results demonstrate that there are lake-to-lake differences in the effects of TP and water color on lake CHL and that this variation is spatially structured. Quantifying spatial structure in these relationships furthers our understanding of the variability in these relationships at macroscales and would improve model prediction of chlorophyll *a* to better meet lake management goals.

## Introduction

A longstanding goal in limnology and lake management is to develop empirical models to predict lake primary production from nutrient concentrations. However, these models can exhibit a great deal of variation in predictive performance across studies. The nutrient-water color paradigm has been proposed to help account for these differences by recognizing that lake trophic condition is characterized by measures of *both* nutrients and water color (the colored component of dissolved organic carbon) in contrast to examining nutrients alone [1–3]. Lake primary production measures, such as chlorophyll *a* (CHL), have been shown to be strongly related to both phosphorus, a limiting nutrient in temperate North American lakes, and water color, a strong light attenuator, but in potentially contrasting directions [1,4–6]. However, because few studies have examined the effects of phosphorus and water color on lake CHL together [1,3], it is unclear how well the nutrient-water color paradigm applies to lakes distributed across diverse landscape settings. Understanding the relative strength of these drivers of lake productivity will be especially important to predict how lakes may respond to ongoing and future global changes that are altering nutrient and carbon inputs to freshwater systems [7–9].

Although the empirical relationship between CHL and lake total phosphorus (TP) is well established, no consensus has been reached about the additional role of water color in lake primary production. Water color can influence lake primary production in complex and confounding ways [1,5,10,11]. The strong light attenuating effects of water color can inhibit photosynthesis and reduce phytoplankton abundance [5,12]. In contrast, water color has also been positively associated with primary production by directly supplying nutrients to aquatic systems. Humic substances can form complexes with nutrients and thus be sources of inorganic nutrients to lakes [13–15]. Water color can also stimulate primary production by indirectly promoting processes that release nutrients. For example, spectral properties of water color can influence the mixing depth in small lakes [16] and subsequently promote biogeochemical conditions to release nutrients from the sediment, which can stimulate primary production [17]. It is difficult to study water color effects across large populations of lakes because the contrasting effects of these different mechanisms may cancel out their overall effect on primary production, and it is likely that these mechanisms operate at different spatial and temporal scales.

There are several lines of evidence that indicate that TP and water color relationships with CHL vary over space and may exhibit spatial autocorrelation such that lakes near one another have more similar relationships compared to lakes that are further away. First, land use and land cover are major sources of both phosphorus and carbon to lakes and these landscape features vary broadly across space. For example, agriculture land use and wetland cover are recognized sources of phosphorus and dissolved organic carbon, respectively, to lakes [18,19]. Second, comparative studies at broad spatial extents demonstrate that the effect of TP on CHL varies across ecological regions such that lakes within regions have more similar TP–CHL relationships compared to lakes from other regions [20,21]. Similarly, dissolved organic carbon relationships with primary production exhibit strong among-region differences [11,20,21]. Finally, other spatially structured features, such as topography, geology, and hydrologic connectivity, may influence the delivery of and in-lake processing of nutrients and carbon [3,6,22] and consequently affect primary production, leading to further spatial structuring of the relationships between driver and response variables. Lake community composition (herbivore assemblages and macrophyte coverage) has been related to differences in TP and CHL relationships [4,5,23], and these biological attributes are likely to exhibit spatial variation that is influenced by dispersal properties [24]. However, data on aquatic community composition are often lacking for multiple lakes distributed over broad spatial extents, and it can be difficult to directly incorporate biological factors in these relationships.

Although it is likely that TP and water color relationships with lake CHL vary spatially, few studies have explicitly examined and accounted for spatial dependencies. Ignoring spatial autocorrelation is problematic because 1) it violates statistical assumptions of independence, which in effect reduces the effective sample size and can lead to falsely identifying covariates as significant, 2) it can exclude relevant covariates form the model, and 3) it produces less accurate prediction estimates [25]. In addition, by allowing for model parameters to vary over space, we can improve inference and gain insight about other potential spatial drivers of these relationships that may not have been measured or considered before [26].

Previous studies accounted for spatial variation in driver and response relationships by using discrete spatial units, such as ecological regions, to partition the landscape into ecologically similar patches and capture variation in lake response variables [6,20,21]. While these discrete spatial units improve model accuracy, they may not optimally delineate the landscape to capture spatial variation in TP–CHL and water color–CHL relationships. For example, variation in these relationships may occur at finer spatial extents than the chosen ecoregion boundaries. In addition, the spatial drivers of TP–CHL relationships may operate at different spatial scales than the spatial drivers of water color–CHL relationships. Thus, confining variation to fixed ecological regional boundaries may not be the way to understand and quantify relationships among TP–CHL and water color–CHL at the macroscale.

In this paper, we explore the nutrient-water color paradigm for over 800 lakes across diverse landscape settings to examine broad-scale spatial heterogeneity in CHL relationships with TP and water color. We ask the following questions, 1) Do TP and water color relationships with CHL vary among lakes at sub-continental scales? And 2) If so, at what spatial scale do these relationships vary [27]? To answer these questions, we use a Bayesian framework and fit spatially-varying coefficient (SVC) models to lakes located in the Midwest and Northeast regions of the U.S. The SVC model allows for regression model intercept and slope parameters (i.e., coefficients) to vary over continuous space rather than among discrete ecological regions. Specifically, each regression coefficient is modeled using a Gaussian process, with mean, variance, and distance correlation decay parameters estimated using a valid probability model. With this modeling approach, we can quantify the spatial range at which spatial dependency in parameter values diminish and identify spatial scales that capture variation in TP and water color relationships separately. We included covariates in the models that have been shown to be related to lake CHL (e.g., lake depth), and we explored whether the lake-specific spatially-varying coefficients were related to hypothesized lake and catchment characteristics using correlation analyses. Quantifying spatial variation in TP and water color relationships with lake CHL at macroscales should improve model inference and provide insight about the relative strength of nutrient and water color drivers of lake primary production.

## Methods

### Lake and landscape datasets

Data used in the analyses come from the LAGOS database (Lake multi-scaled geospatial and temporal database [28]). LAGOS is a multi-thematic lake database that integrates lake water chemistry data (LAGOS_LIMNO_) and geospatial data (LAGOS_GEO_) across the U.S. Midwest and Northeast regions. We accessed LAGOS_LIMNO_ version 1.040.0 and LAGOS_GEO_ version 1.02 for this study.

**Fig 1:**
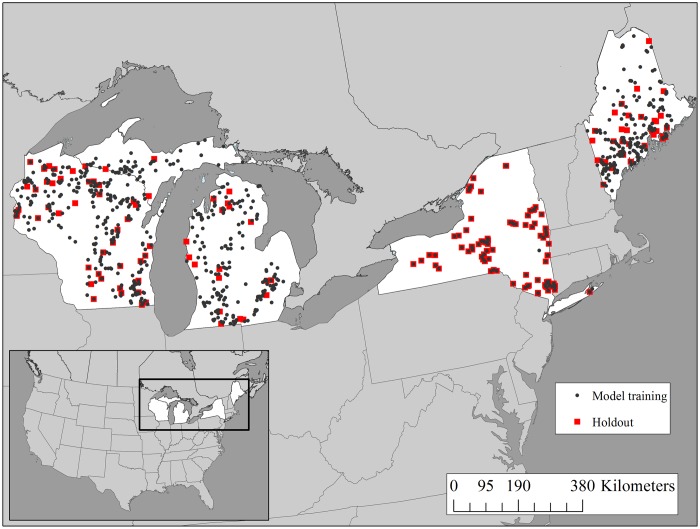
Study extent map. Lake locations in the analysis (N = 838 lakes) including model training observations (n = 6656) and locations of holdout observations for model predictive performance (n = 739). Lakes in New York were sampled through time and thus some observations were part of the model training dataset and other observations were part of the model predictive performance holdout dataset.

We used a subset of lakes from the LAGOS dataset with water chemistry and lake geomorphology data related to our research questions. Our dataset included lakes (greater than 4 ha and less than 10,000 ha in surface area) that had summer (15 June– 15 September) epilimnetic CHL, TP, and water color observations measured during the same sampling event. We omitted lakes missing maximum depth data from our analysis because lake depth is recognized to affect nutrient processing and primary production, making it an important variable to include in the models. In total, the data included 838 lakes (7395 observations) within Wisconsin (WI), Michigan (MI), New York (NY), and Maine (ME) (Fig 1) and captured a wide range of lake and catchment characteristics (Table 1). Lakes in the dataset were spatially distributed with the median distance of a lake to it’s nearest neighbor being ~6 km (range = 0.4–72 km). Data and metadata for this study are available at the Long-Term Ecological Research Network Data Portal doi: 10.6073/pasta/0ebd2e4c0705706b77b359955bff44e1 [29].

**Table 1:** Summary statistics of the full lake dataset.

Variable	Mean	Median	Range	Standard deviation
Chlorophyll *a* (μg/L)	10.72	4.47	0.01–363.00	19.23
TP (μg/L)	21.97	14.00	0.90–494.00	27.07
Water color (PCU)	20.30	14.00	1.00–194.00	21.05
Max. depth (m)	11.54	9.20	1.52–58.50	8.23
Lake area (ha)	230.00	55.49	4.28–7043.36	578.38
Catchment area (ha)	4976.00	654.70	3.90–436923.90	19394.01
CA:LK	26.06	10.06	0.27–7444.23	113.32
Prop. Agriculture	0.17	0.10	0–0.84	0.18
Prop. Urban	0.10	0.05	0–0.96	0.15
Prop. Wetland	0.10	0.06	0–0.81	0.11
Prop. Forest	0.10	0.06	0–0.63	0.10

The mean, median, range, and standard deviation of lake water chemistry, lake geomorphology, and landscape variables for the full dataset (n = 7395 observations, N = 838 unique lakes). Prop. = proportion in the lake catchment. CA:LK = catchment to lake area ratio.

Lake CHL, TP, and water color data were collected by state agencies from 1986–2013 following standard field collection and laboratory methods. The majority of lakes in the dataset (>70%) have a single water chemistry observation over time. There are several lakes (N = 228) with multiple observations over time, and these are mainly located in New York (S1 Fig). Lakes with multiple observations had, on average, about 30 observations, with most of the observations occurring across years (i.e., not within the same season each year). We checked for temporal autocorrelation in water chemistry measurements for individual lakes by examining residual plots over time and did not find evidence for either among-year trends or within-year (seasonal) trends that would need to be accounted for in the model design. Thus, we kept multiple water chemistry observations per lake over time in the dataset to increase the number of observations used to fit the models.

We related lake CHL and spatially-varying coefficients to lake hydrogeomorphology and catchment variables in LAGOS_GEO_. In LAGOS_GEO_, lakes were assigned a hydrologic connectivity type based on the presence or absence of surface stream connections represented in the National Hydrography Dataset (NHD) (see [28] for methods used to identify lake hydrologic type). Lakes were identified as either *isolated* (i.e., no inflowing streams; N = 232 with 1499 observations) or *drainage* (i.e., inflowing streams; N = 606 with 5896 observations). Mean and standard deviation values for lake and catchment variables by lake type are available in S1 Table. Catchment boundaries were delineated for each lake in the study extent using automated Geographic Information Systems (GIS) methods (LAGOS GIS Toolbox [28]). Land use and land cover class proportions within the lake catchments were quantified from the 2006 National Land Cover Database because the majority of water chemistry data were collected around this year.

### Analysis

#### Model framework overview

We applied SVC models within a Bayesian inferential framework to examine variation in TP and water color relationships with CHL over space. SVC models are suited to our research questions because they allow for the explicit examination of both space-varying and scale-dependent relationships between nutrient and color drivers and lake chlorophyll *a*. SVC models allow for selected model regression coefficients to vary by point locations and produce smoothly varying coefficient surfaces that are modeled as realizations from spatial processes [30]; therefore, the models do not assume that coefficients are stationary (i.e., constant) over space—allowing for inference about location-specific effect of drivers on the response. In contrast to multi-level mixed effects models that use discrete areal units to model spatial dependency, SVC models allow for greater flexibility and relieve the constraint of modeling variation among potentially arbitrarily-specified areal units that may not optimally capture the scale of spatial variation across the different covariates. The Bayesian framework produces posterior probability distributions that allow for full uncertainty quantification in parameter estimates and subsequent predictions at unobserved locations within the domain.

#### Model description

SVC model structure took on the following form. We model log CHL *y*_*t*_(***s***) at lake location ***s*** and sample time *t* as

$$y_{t}\left(s\right)=\;{\tilde{x}}_{t}{\left(s\right)}^{\text{T}}\;\tilde{\beta }\left(s\right)+\;x_{t}{\left(s\right)}^{\text{T}}\;\beta +\;e_{t}\left(s\right)$$

Where ${\tilde{x}}_{t}\left(s\right)$ is an intercept with lake and time specific measurements of log TP and log water color, i.e., ${\tilde{x}}_{t}\left(s\right)={\left(1,\;TP_{t}\left(s\right),\;color_{t}\left(s\right)\right)}^{\text{T}}$, and $\tilde{\beta }\left(s\right)$ is the associated vector of spatially-varying regression coefficients. Additional covariates with spatially invariant regression coefficients are specified in ***x***_*t*_(***s***) and ***β***, respectively. Model residuals are assumed to follow a zero-centered normal distribution that is independent across measurement location and time, i.e., *e*_*t*_(***s***) ~ *N*(0, *τ*^2^) where, *τ*^2^ is the residual variance parameter that captures measurement error. We assume $\tilde{\beta }\left(s\right)$ follows a multivariate Gaussian process, i.e., $\text{MVGP }\left({\tilde{\beta }}_{mu},\;\sum \left(\theta \right)\right)$ where ${\tilde{\beta }}_{mu}$ is the mean regression coefficients over the domain and Σ(***θ***) is the covariance matrix with ***θ*** including an intercept, TP, and water color specific spatial correlation decay parameters (*φ*) and cross-covariance parameters. The MVGP is constructed using a Linear Model of Coregionalization (see, e.g., [27]).

We quantified the distance at which the spatial dependence in model coefficient values becomes negligible by calculating the effective spatial range. The effective spatial range is based on the spatial correlation decay parameters (*φ*). We define the effective spatial range as the distance at which the spatial correlation drops to 0.05 between observations [26]. The effective spatial range provides an estimate of the spatial scale that captures variation in lake TP and water color effects on CHL.

We evaluated four hypothesized candidate models to examine the potential spatially structured effects of TP and water color on lake CHL. The first candidate model was a non-spatial linear regression relating TP and water color to CHL that estimated spatially-invariant, global model coefficients. The second model (SVC_TP,COLOR_) allowed the intercept, TP, and water color regression coefficients to vary spatially. The third model (SVC_LANDSCAPE_) had the same spatially varying coefficients (i.e., intercept, TP, and water color) and also included hypothesized lake (maximum depth, catchment to lake area ratio—CA:LK) and landscape (proportion agriculture and wetland area in the catchment) space invariant covariates. These covariates were included in the models because they have been related to lake primary production and water chemistry concentrations in the literature [3,31,32] and they did not exhibit strong collinearity with one another. The importance of model covariates to predict CHL were based on coefficient 95% credible intervals not overlapping zero. The fourth model (SVC_FULL_) built upon the SVC_LANDSCAPE_ model by including a dummy variable to identify the lake connectivity type (*isolated* vs. *drainage*). We log_10_ transformed CHL, TP, water color, maximum depth, and CA:LK to reduce skewness of the data.

#### Model evaluation and predictive performance

The candidate non-spatial and SVC models were evaluated two ways: (1) model fit to the data and (2) predictive performance using out-of-sample cross-validation. Prior to model building, 90% of the observations (n = 6656) in the dataset were selected at random and used to estimate candidate models’ parameters, and the remaining 10% of observations (n = 739) were withheld to evaluate model predictive performance. To evaluate the fit of the candidate models to the observed data, we used the deviance information criterion (DIC), an information criterion that can be used to compare models that apply a Bayesian framework [33]. DIC is calculated as the sum of the Bayesian deviance value (D) and estimated effective number of parameters in the model (pD), where lower DIC values indicate better model fit. For the out-of-sample cross-validation, the parameter posterior samples for the model-fitting dataset were used to generate posterior predictive samples for the holdout observations [34]. Then, using the holdout observations and model posterior predictive distribution samples, predictive performance was summarized using 1) root mean-square prediction error (RMSPE) between observed values and means of the predictive distributions; 2) mean continuous rank probability score (CRPS), which is a strictly proper scoring rule that quantifies the fit of the entire predictive distribution (i.e., for a normal distribution, the mean and the variance) to the data [35]; 3) percent of observations covered by their corresponding predictive distribution 95% credible interval (PCI) and mean width of the predictive distributions' 95% credible interval (PIW). Lower values of RMSPE and CRPS indicate better predictive performance. Similarly, we favored models that provided narrow posterior predictive interval widths (PIW) while delivering appropriate posterior coverage rates, i.e., PCI at ~90%.

Finally, we explored whether spatial variation in TP and water color relationships were related to underlying lake and catchment characteristics using Pearson correlation analyses. We related the mean posterior coefficient values (i.e., lake specific intercept, TP, and water color slopes) estimated from the SVC_TP,COLOR_ model (the model that did not include any of the spatially-invariant lake and landscape covariates) to hypothesized lake and catchment variables. Mean differences in spatially-varying coefficient values among lake connectivity types were assessed using Welch two-sample t-tests. Correlation and t-test analyses were performed using base packages in R statistical platform (R Core Team 2015). The non-spatial model was run in R version 3.1.2 using spBayes statistical package [36]. SVC models were written and compiled in C++ programming language and R.

## Results

The SVC models that included spatially-varying intercept, TP, and water color coefficients were better models in terms of fit and predictive performance compared to the non-spatial model. Among the SVC models, the top ranked model was SVC_FULL_ that included spatially-varying intercept, TP, and water color coefficients in addition to spatially-invariant (i.e., fixed) lake and landscape coefficients (Table 2). The DIC and D values were the lowest for SVC_FULL_ compared to the other candidate models, indicating a better model fit to the observed data despite the model being penalized for including additional parameters. In terms of model predictive performance, the SVC models performed similarly well and provided improved RMSPE and CRPS over the non-spatial regression and acceptable coverage rates with PCI greater than 90% and narrower PIW (Table 2).

**Table 2:** Summary of TP and water color ~ CHL candidate models including posterior estimated coefficients, model fit criteria, and model predictive performance measures.

	Non-spatial	SVC_TP,COLOR_	SVC_LANDSCAPE_	SVC_FULL_
Intercept $\left({\tilde{\beta }}_{0}\right)$	-1.12	-0.43	-0.34	-0.36
	(-1.20, -1.04)	(-0.57, -0.29)	(-0.61, -0.08)	(-0.61, -0.10)
TP $\left({\tilde{\beta }}_{TP}\right)$	1.06	0.73	0.71	0.698
	(1.03, 1.09)	(0.67, 0.79)	(0.64, 0.77)	(0.63, 0.77)
Color $\left({\tilde{\beta }}_{Color}\right)$	-0.06	-0.002	-0.01	-0.02
	(-0.09, -0.04)	(-0.086, 0.094)	(-0.10, 0.07)	(-0.10, 0.08)
Z_MAX_ (*β*_*Zmax*_)			-0.08	-0.13
			(-0.16, -0.01)	(-0.19, -0.04)
CA:LK (*β*_*CALK*_)			0.05	0.02
			(0.01, 0.09)	(-0.03, 0.07)
AGR (*β*_*AGR*_)			0.52	0.45
			(0.18, 0.83)	(0.13, 0.77)
WET (*β*_*WET*_)			0.29	0.16
			(-0.25, 0.87)	(-0.38, 0.73)
Drain. (*β*_*Drain*_)				0.21
				(0.08, 0.34)
*τ*^*2*^	0.82	0.63	0.63	0.63
	(0.79, 0.85)	(0.60, 0.65)	(0.61, 0.65)	(0.61, 0.65)
Eff. Range _Intercept_		21.78	14.21	32.56
		(19.33, 26.37)	(12.81, 15.71)	(19.90, 119.15)
Eff. Range _TP_		19.98	33.75	26.32
		(17.91, 23.58)	(27.47, 39.66)	(16.78, 99.41)
Eff. Range _Color_		302.07	442.43	216.05
		(199.53, 443.56)	(311.81, 537.24)	(138.98, 276.62)
ΔDIC	1457.09	16.65	27.75	0
pD	3.94	324.54	310.41	322.08
D	10887.70	8348.76	8388.56	8329.50
RMSPE	0.88	0.77	0.77	0.77
CRPS	0.48	0.42	0.42	0.42
95% PCI	94.74	90.68	90.23	90.25
95% PIW	3.52	2.51	2.48	2.45

Model coefficient posterior means are presented with 95% credible intervals. The residual variance parameter (*τ*^*2*^) quantifies measurement error. The effective spatial range values (km) are calculated for the spatially-varying coefficients based on spatial decay parameters *φ*_1_, *φ*_2_, *φ*_3_. Models are ranked based on deviance information criteria (DIC) scores where lower values indicate a better model fit. The effective number of parameters (pD) are taken into account in the DIC scores (based on Bayesian deviance value D) to penalize more complex models. Model predictive performance is summarized using root mean-square predictive error (RMSPE), mean continuous rank probability score (CRPS), percent of observations covered by their corresponding predictive distribution 95% credible interval (PCI), and mean width of the predictive distributions’ 95% credible interval (PIW). Smaller RMSPE and CRPS values indicate better predictive performance, larger PCI values indicate increased model accuracy, and smaller PIW indicate increased precision. Z_MAX_ = maximum lake depth, CA:LK = catchment to lake area ratio, AGR = proportion agriculture in lake catchment, WET = proportion wetland in lake catchment, and Drain. = lake connectivity type category.

### Lake and landscape covariates modeled as spatially-invariant (i.e., fixed across lake location)

The top-ranked model based on DIC values included spatially-varying coefficients and lake and landscape variables that were modeled as spatially-invariant, or to have fixed effects across locations. The lake and landscape covariates that were important in the model had expected relationships with CHL (Table 2). Maximum lake depth was negatively associated with CHL—such that deeper lakes tended to have lower CHL concentrations in comparison to more shallow lakes. Proportion of agricultural activity in the catchment and lake connectivity type were positively associated with CHL. Lakes with high agricultural land use in their catchments had higher CHL concentrations compared to lakes with less agricultural activity. Including a dummy variable to indicate lake connectivity type indicated that drainage lakes had higher CHL compared to isolated lakes. Catchment area to lake area ratio (CA:LK) and proportion of wetland cover in the catchment showed no discernable relationship with CHL. It should be noted that the lack of relationships in this model does not necessarily mean that these covariates are not important; the effect of these covariates may vary across locations in our study extent and may be difficult to detect.

### Spatially-varying model coefficients

#### Spatially-varying intercept

**Fig 2:**
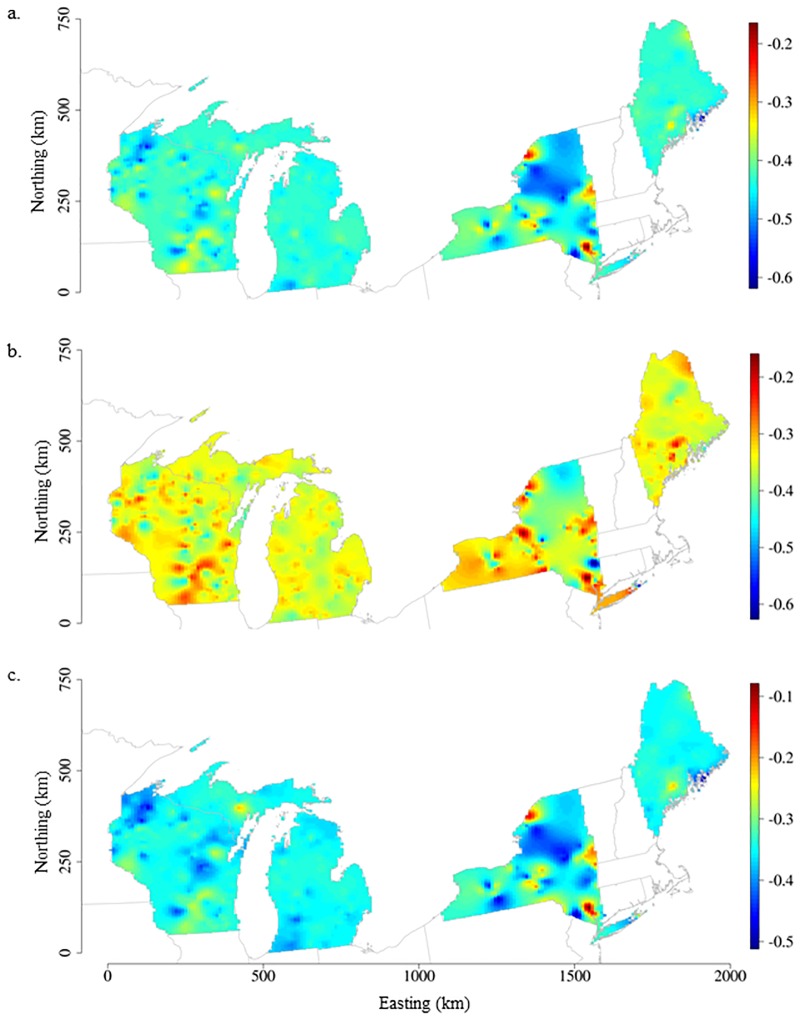
Spatially-varying intercept surface maps for a) SVC_TP,COLOR_, b) SVC_LANDSCAPE_, and c) SVC_FULL_ models. Interpolated surface maps were derived from the posterior mean of the spatially-varying intercept values estimated by lake location in the model building dataset (N = 779) and displayed as blue to red color gradients representing low to high intercept values.

Spatially-varying intercepts and TP and water color effects (i.e., slopes) on CHL improved model fit compared to the non-spatial, regression model. This improvement indicated that in addition to the effects of phosphorus and water color on lake chlorophyll *a*, lake CHL concentrations exhibited spatial autocorrelation across this diverse set of north temperate, inland lakes. Allowing for the model intercept values to vary across lake locations captured spatial autocorrelation in lake CHL that was not accounted for by the mean function and subsequently helped meet the model assumptions that residuals are independent and identically distributed. Lake and landscape covariates added to models SVC_LANDSCAPE_ and SVC_FULL_ reduced and smoothed remaining spatial variation in lake CHL as seen in the spatially-varying intercept surface maps (Fig 2).

#### Spatially-varying TP and water color effects on CHL (i.e., spatially-varying slopes)

**Fig 3:**
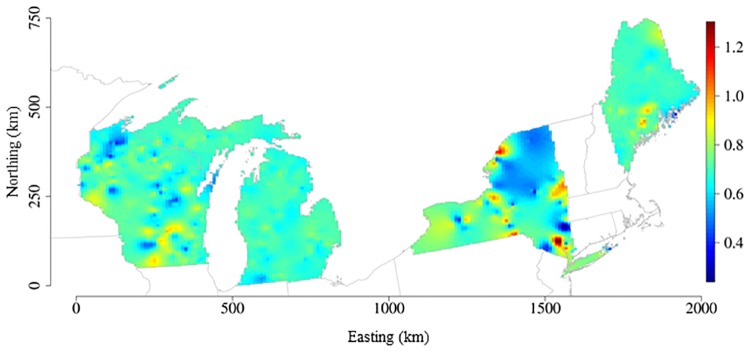
Spatially-varying TP–CHL coefficients maps derived from the SVC_FULL_ model. Surface map of spatially-varying TP–CHL relationships created by interpolation of the posterior mean values that were estimated by lake location in the model building dataset (N = 779). Blue to red color gradient represents low to high TP–CHL coefficient values.

The SVC_FULL_ model estimated the mean effects of TP and water color on CHL; however, it was more informative to examine the spatially-varying coefficients across locations rather than the global, mean effects to better understand the distribution of these coefficient values over the study extent. The spatial processes that captured variation in model coefficients were different for TP compared to water color, suggesting that there may be different underlying factors that influence TP and water color effects on CHL. Lake TP was positively related to CHL for all lakes, but the magnitudes of these effects were different across locations (Fig 3). The posterior mean log CHL–log TP coefficient for the study lakes was 0.73 (±0.84) and ranged from 0.27 to 1.38 (S2 Fig). Translating these values into effects on CHL, on average 1 μg/L increase in TP was related to 5 μg/L increase in lake CHL. However, TP~ CHL effects were variable across individual lakes with a 1 μg/L increase in TP resulting in increased CHL ranging from 2–24 μg/L.

**Fig 4:**
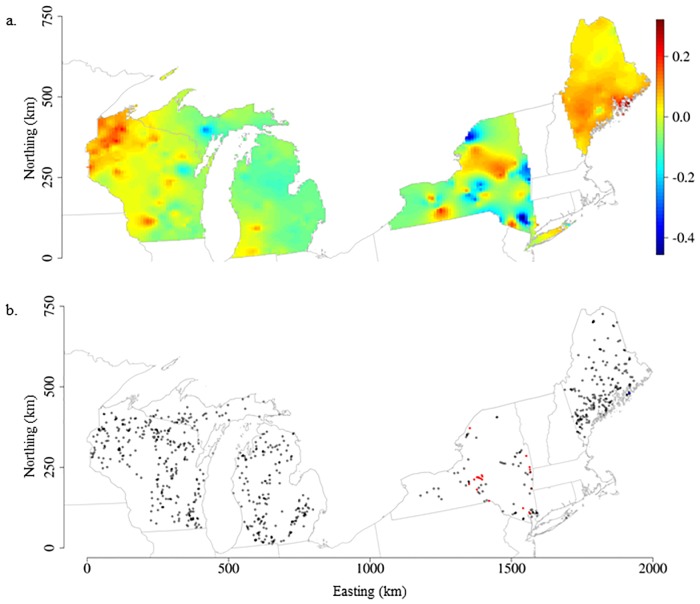
Spatially-varying water color–CHL coefficients maps derived from the SVC_FULL_ model. a) Surface map of spatially-varying water color–CHL relationships created by interpolation of the posterior mean values that were estimated by lake location in the model building dataset (N = 779). Blue to red color gradient represents low to high water color–CHL coefficient values. b) Map of lake point locations symbolized by water color–CHL relationships: positive (blue), negative (red), not significant (black outlined dot). Significant relationships were determined based on 95% credible intervals not overlapping zero.

In contrast, water color effects on CHL varied over space but were not important for the majority of lakes in the study extent (Fig 4). Where water color effects were significant, some lakes had positive water color relationships with CHL (mean SVC_Color_ 0.30 ± 0.02; N = 4 lakes) and other lakes had negative water color relationships with CHL (mean SVC_Color_ -0.26 ± 0.07; N = 16 lakes) (S2 Fig). The lake with a maximum positive water color effect on CHL resulted in a 2.14 μg/L increase in CHL per unit increase in water color. The lake with the greatest negative water color effect resulted in a 2.67 μg/L decrease in CHL per unit increase in water color.

The scales at which coefficients exhibited spatial autocorrelation were different for TP–CHL effects compared to water color–CHL effects. In the SVC_FULL_ model, the effective spatial range for TP–CHL was 26 km (Table 2), indicating that lakes within 26 km of one another had more similar TP relationships with CHL compared to lakes that were further away. In contrast, the effective spatial range for water color–CHL was 216 km (Table 2). Because only twenty lakes had significant water color relationships with CHL, this large effective spatial range indicates that there are broad spatial areas where lakes had weak to non-existent water color relationships with CHL (Fig 4b).

Once we established that spatially-varying coefficients improved the model fit, we examined whether hypothesized lake and landscape variables were related to spatial variation in these effects using the spatially-varying coefficients from the SVC_TP,COLOR_ model. Lake-specific TP–CHL coefficients were not strongly correlated with maximum lake depth or catchment characteristics (r <0.5; Table 3). There was a statistically significant difference in the mean TP–CHL coefficient values between drainage and isolated lakes (two sample t-test: t = 2.55; df = 441.31; p-value < 0.05); but the difference in mean TP–CHL effects between lake types was small and does not appear to be ecologically meaningful (S3 Fig).

**Table 3:** Correlation coefficient values for lake-specific spatially-varying coefficients and hypothesized lake and catchment variables.

	SVC_INTERCEPT_	SVC_TP_	SVC_COLOR_
log_10_-Secchi	**-0.30**	**-0.38**	**0.20**
log_10_-Zmax	**-0.11**	**-0.14**	**0.15**
log_10_-CALK	**0.12**	**0.12**	**-0.08**
AG	**0.13**	**0.15**	**-0.08**
WET	0.01	0.01	**-0.13**

Spatially-varying intercept (SVC_INTERCEPT_), TP (SVC_TP_), and water color (SVC_COLOR_) coefficients were estimated for 779 lakes from the SVC_TP,COLOR_ model. Significant correlation coefficients (α < 0.05) are in bold.

Similarly, lake-specific water color–CHL coefficients were not strongly correlated with any of the hypothesized lake and catchment characteristics (r <0.5; Table 3). There was a statistically significant difference in the mean water color–CHL coefficient values between drainage and isolated lakes (two sample t-test: t = 2.40; df = 428.40; p-value < 0.05); but the difference was small and may not be ecologically important (S3 Fig).

## Discussion

Our results demonstrate that lake water chemistry relationships with primary production measures (i.e., TP–CHL and water color–CHL) exhibit potentially important lake-to-lake differences that are spatially structured at broad extents. Modeling spatial autocorrelation in TP and water color relationships improved inference (based on DIC scores) and prediction (based on RMSPE) over the model that ignored spatial dependency and provided insight about the spatial characteristics of these relationships. Total phosphorus effects on CHL varied across lakes such that a 1 μg/L increase in TP resulted in increased CHL ranging from 2–24 μg/L. Whereas water color effects on CHL were not important for the majority of lakes, but there were some lakes where water color was positively related to CHL such that a 1 unit increase in water color resulted in an increase in CHL by 2 μg/L; and there were other lakes where water color was negatively related to CHL such that a one unit increase in water color resulted in decreased CHL ranging from 1.4–2.6 μg/L. The scales that capture spatial autocorrelation were different for TP–CHL relationships compared to water color–CHL relationships. Specifically, variation in TP effects on CHL was structured at a more local scale (~20 km), which means that lakes within a 20 km radius had similar TP–CHL relationships. In contrast, variation in water color effects was structured at a more regional scale (~200 km). This may be the first study to examine spatial variation over continuous space of the well-recognized lake TP–CHL relationship and the highly variable water color–CHL relationship. Our results further scientific understanding of the multi-scaled structure of nutrient and water color relationships that control lake primary production (i.e., the nutrient-color paradigm); and they offer insight into identifying appropriate spatial scales for limnological research and water resource management.

### Lake variation in TP–CHL relationships at macroscales

We found that TP–CHL relationships exhibited a great deal of spatial variation in our study extent. The lake-specific log TP–CHL slopes (0.27–1.38) are within the range of values reported in the literature [6]. Several studies have tried to improve TP–CHL predictions by evaluating sources of variation in these relationships, but few studies have examined spatial variation in TP–CHL relationships [6,20,21]. Wagner et al. (2011) found regional differences in TP–CHL relationships within ecological drainage units (EDU) that range in area from 1,000 km^2^ to 10,000 km^2^ [37], such that lakes within regions had more similar TP effects compared to lakes from other regions. In another study, no regional differences in TP–CHL relationships were detected among coarsely delineated regions following country political boundaries for European lakes [6]. This lack of any regional relationship may be because the regions spanned multiple countries in the European Union and captured a great deal of within-region heterogeneity. Our lake-specific TP–CHL relationship results suggest that variation in these relationships occurs at intermediate spatial scales between lake catchment and commonly used ecological region extents. In fact, our results suggest that regional delineations that are ~400 km^2^ in area may more optimally capture variation in TP–CHL relationships over space compared to larger regional extents.

We hypothesized that the spatial variation in TP–CHL relationships estimated from the SVC models would be related to lake geomorphic and catchment characteristics [38]. However, we did not find evidence for any strong associations. These surprising results may be due to scale differences in the selected response and the predictor variables [39]. The spatial scales that landscape variables were quantified (i.e., catchment scale) were not aligned with the spatial scales of variation in TP–CHL relationships (~ 20 km). Alternatively, differences in TP effects may be influenced by complex, cross-scale interactions whereby features at one spatial scale may interact with features at another scale [40–42]. In fact, there is evidence for cross-scale interactions being associated with differences in TP–CHL relationships in other studies. Regional percentage of pasture land was associated with among-region differences in TP relationships with CHL, illustrating an example of features at one spatial scale (i.e., region) interacting with processes at another spatial scale (i.e., lake) [20]. Similarly Filstrup et al. (2014) found that the percentage of pasture and wetlands within the region were related to TP–CHL effects modeled as nonlinear relationships [21]. These findings suggest that there may be broader landscape features beyond the lake catchment that structure differences in TP effects on CHL. Thus, a multi-scaled perspective is warranted.

At the opposite end of the spatial continuum, the variation observed in TP–CHL relationships across the study lakes may be related to a number of in-lake characteristics such as differences in morphology, water chemistry, and zooplankton and macrophyte community composition [4,6,23,43]. We did not find support that maximum lake depth was associated with spatial differences in TP–CHL relationships. However, spatial differences in these relationships may be related to unmeasured water chemistry variables and lake community composition characteristics that are linked to landscape sources and spatial dispersion factors [24,44].

Total nitrogen to total phosphorus ratios (TN:TP) and alkalinity have been associated with variation in TP–CHL relationships and are tightly linked to land use activity and geological composition in the landscape. Lakes with very low TN:TP ratios have relatively weak TP–CHL relationships due to N-limitation [45,46]. The form of agricultural activity can also influence TN:TP ratios. For example, row-crop activity is associated with high TN:TP ratios and pasture is associated with low TN:TP ratios [47]. In our study, total nitrogen data were not available for most of our study lakes, but we distinguished between agriculture NLCD classes (cultivated land vs. pasture) and used these classes as indicators of nutrient ratios exported to lakes. We did not see a strong correlation between agriculture type and lake-specific TP effects (cultivated land r = 0.11 and pasture r = 0.17). However, it should be noted that the accuracy of agricultural land use class specification is not fool-proof [48]. Alkalinity of lakes has been associated with decreased chlorophyll *a* yield per unit of phosphorus due to phosphorus precipitating out of solution [49], other studies show no strong association among geological indicators of alkalinity and variation in TP–CHL [20]. We lacked data on alkalinity for our study lakes to properly explore this relationship, but it is worth investigating in future studies.

Lake community composition has also been related to differences in TP–CHL relationships. Large zooplankton herbivore communities have been associated with lower CHL yields per unit TP across different lake trophic classes [4]. And increased macrophyte coverage was associated with lower lake chlorophyll *a* production [23]. Macrophyte and zooplankton community composition in lakes may be structured by spatial factors that influence dispersal such as hydrologic connectivity [24] and may be related to spatial variation in TP–CHL relationships. However, we did not have lake community composition data for our study lakes, and it may be that these spatial factors would operate at finer spatial scales than the intermediate spatial scales observed.

### Lake variation in water color–CHL relationships at macroscales

Although most lakes did not exhibit significant water color effects on CHL, there were four lakes that had positive water color effects (log slope range 0.28–0.34) and sixteen lakes that had negative effects (log slope range -0.42 –-0.15) with large ecological implications for lakes. It was not surprising that water color effects on lake chlorophyll *a* were not significant in the global model because these contrasting positive and negative relationships cancel one another out. However, we expected to find more individual lakes with significant color effects than what was observed. The results suggest that water color effects on lake CHL may be less important compared to TP effects for north temperate lakes in areas with mixed land use/cover. However, lake DOC and water color concentrations are shown to exhibit regional patterns that are related to underlying landscape and climatic features [41,50,51]. In addition, in northern boreal and arctic lakes, dissolved organic carbon is shown to have a nonlinear relationship with lake primary productivity such that at low concentrations DOC is positively associated with primary production (acting as a nutrient source by carrying P) and at high concentrations it is negatively associated with primary production by inhibiting light availability [11,52]. Therefore, regional patterns of lake organic carbon coupled with nonlinearities in DOC relationships with primary production may account for the lack of a strong water color relationship in our study extent. Additionally, our study lakes did not capture a wide range of water color (1–194 PCU) and the distribution was skewed towards low colored lakes (<20 PCU). Thus, these facts might have contributed to our weak color—CHL results, especially in disturbed landscapes where there are more prolific landscape nutrient sources (e.g., agriculture).

There were too few lakes with significant water color relationships to draw definitive conclusions on what promotes differences in water color effects on CHL. However, we describe the general characteristics of these lakes to identify potential lake and catchment variables to explore in future studies. Lakes that exhibited significant positive water color–CHL relationships were deep, oligotrophic lakes with catchments dominated by forest cover and minimal human disturbances (S2 Table). These lakes had moderate wetland cover in their catchments (0–2%), but the majority of wetland patches were connected to streams in the catchment, suggesting a potential mechanism of carbon transport to the lake [53]. In contrast, lakes that exhibited significant negative water color–CHL relationships tended to be less deep, mesotrophic lakes with moderate levels of agricultural land use in the catchment. These patterns suggest that land use disturbance may influence the relationship between water color and lake chlorophyll *a*.

### Lake variation in chlorophyll *a* at macroscales

Allowing lake CHL (i.e., model intercept) to spatially vary by lake improved the model fit to the observed data. Even after accounting for TP and water color effects, lake CHL exhibited spatial heterogeneity at intermediate scales with an effective range of ~30 km (Table 2). This indicated that lakes that are within 30 km of one another have more similar CHL compared to lakes that are located further away and that there may be underlying spatially-structured variables that promote these patterns of CHL. We found that lake and catchment predictor variables included in the top-ranked model improved model fit of observed CHL and accounted for some of the spatial variation in lake CHL (i.e., model intercepts), indicating that these predictor variables exhibit spatially heterogeneity that promoted the spatial patterns of CHL observed in the study lakes. These variables followed expected relationships with CHL; maximum lake depth had a negative effect on CHL and proportion agricultural land use and lake connectivity type had a positive effect on CHL.

Lake depth is recognized as an important lake geomorphological characteristic that influences in-lake physical, chemical, and biological processes such as mixing regime, water residence time, and nutrient dynamics [54]. Deep lakes tend to have lower total phosphorus and water color concentrations compared to shallow lakes [3,19,55], which can lead to lower primary production. While lake depth does not appear to exhibit strong spatial autocorrelation at broad spatial extents [56], topographic features in the surrounding landscape are related to lake depth [57,58], suggesting that it may exhibit some spatial structure related to the spatial variation observed in CHL for our study lakes.

The proportion of agricultural land use in the catchment was positively associated with CHL. Agricultural land use is a recognized nonpoint nutrient pollution source to lakes that can subsequently influence primary production in lakes [59]. Agricultural activities in the landscape exhibit non-random spatial patterns related to underlying topographical and geological features that constrain locations of land use change [60]. Additionally, nutrient loadings to the catchment from different agricultural practices have been shown to exhibit distinctive spatial heterogeneity [61]. Together these spatial characteristics of agricultural land use may account for the observed lake CHL spatial patterns.

Lake connectivity type was related to CHL such that drainage lakes had higher concentrations of CHL compared to isolated lakes. The two lake connectivity groups had distinguishing lake and catchment characteristics that may promote differences in primary production. Drainage lakes had larger catchments compared to isolated lakes (median = 1355.91 ha vs. median = 200.71 ha) and a greater amount of agriculture in the catchment compared to isolated lakes (median = 6% vs. median = 3%), which may promote differences in CHL concentrations among lake types.

We did not find significant relationships for either CA:LK nor the proportion of wetlands in the catchment with CHL. CA:LK may not capture important lake-landscape processes that influence primary production. It was not surprising that there was no relationship between wetland cover and lake CHL. Wetlands have complex relationships with nutrient dynamics and primary production in lakes such that they can have confounding effects among lakes across broad spatial extents [41]. Although we did not allow for wetland effects to vary by lake because our main focus was on evaluating differences in TP and water color effects over space, modeling wetland effects as a global mean effect may not be appropriate for future macroscale analyses due to regional differences in the effects of wetlands on lakes.

It should be noted that there was remaining spatial variation in lake CHL that was not accounted for by the predictors included in the model. However, the effective spatial range for the model intercept (~ 30 km) can assist in identifying potential landscape variables that are structured at similar scales and may account for CHL variation.

In conclusion, quantifying spatial structure in TP and water color effects on chlorophyll *a* helps to expand our understanding of the spatial variability in these relationships that define the nutrient-color paradigm, over broad spatial extents and diverse lake types. As more focus turns toward adopting macroscale frameworks to address global change at broad scales, there is a need for innovative analytical approaches that can allow for spatial dependencies in such data. SVC models are one approach to improve model prediction and quantify spatial scales of variation in complex ecological relationships.

## Supporting Information

S1 Fig Map of study extent and distribution of number of water chemistry observations by lake. Lakes are symbolized by number of water chemistry observations with gray representing single observations and a yellow to red color gradient representing multiple water chemistry observations by lake.
(TIF)

S2 Fig Histograms of spatially-varying coefficient values. Distributions of a) log TP–CHL and b) log water color–CHL spatially-varying coefficients estimated in the SVC_TP,COLOR_ model.
(TIF)

S3 Fig Boxplots of spatially-varying coefficient values by lake connectivity type. The distributions of spatially-varying coefficient values estimated in the SVC_TP,COLOR_ model for a) spatially-varying intercept; b) TP; c) and water color coefficient values by lake connectivity type. Lakes are identified as drainage (i.e., presence of inflowing streams; N = 566 lakes) or isolated (i.e., no inflowing streams; N = 213 lakes). Mean values among lake connectivity types are significantly different from one another based on t-tests (α < 0.05).
(TIF)

S1 Table Mean and standard deviation values by lake connectivity type. Mean and standard deviation (sd) values for lake water chemistry and lake and catchment characteristics were quantified by lake connectivity type in the full dataset (N = 838 lakes).
(DOCX)

S2 Table Characteristics of lakes with positive and negative water color–CHL relationships. Summary statistics on lake water chemistry variables and hypothesized lake and landscape covariates for lakes with significant positive water color–CHL relationships (N = 4 lakes) and significant negative relationships (N = 16 lakes). Prop. = proportion in the lake catchment.
(DOCX)
