# Supplementary figures and images for: Spatial Variation in Nutrient and Water Color Effects on Lake Chlorophyll at Macroscales
Source: PLoS One. 2016 Oct 13;11(10):e0164592. doi: 10.1371/journal.pone.0164592 (PMC5063324; doi:10.1371/journal.pone.0164592)

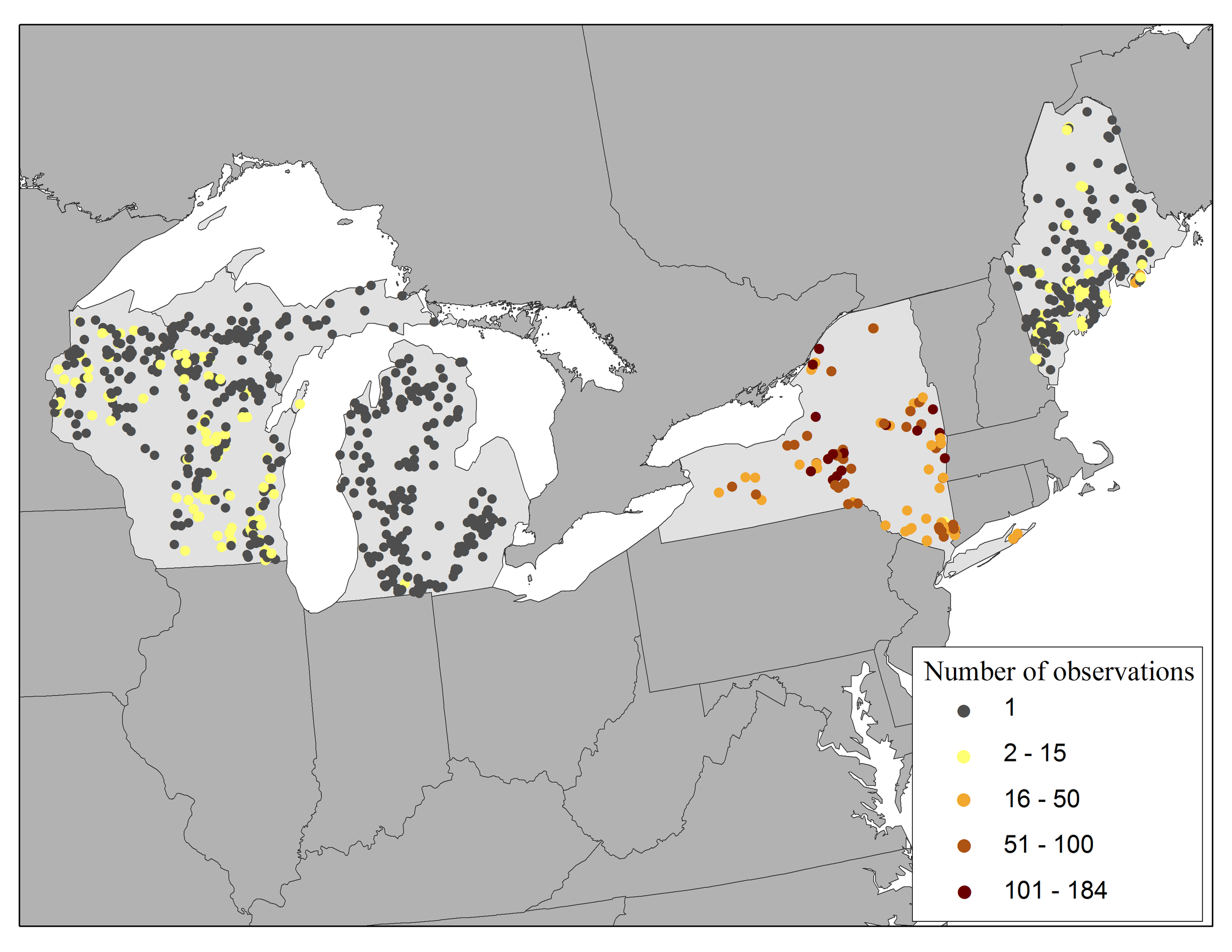

Supplement: S1 Fig — Lakes are symbolized by number of water chemistry observations with gray representing single observations and a yellow to red color gradient representing multiple water chemistry observations by lake. (TIF) [file pone.0164592.s001.tif]

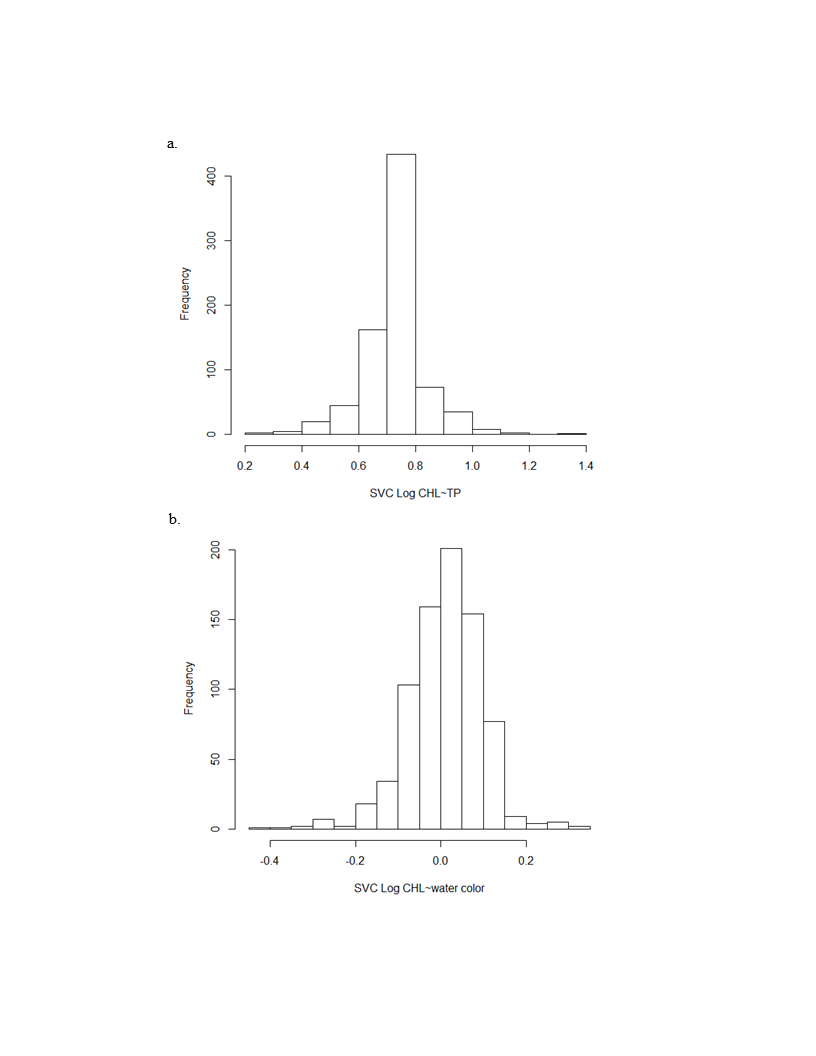

Supplement: S2 Fig — Distributions of a) log TP–CHL and b) log water color–CHL spatially-varying coefficients estimated in the SVCTP,COLOR model. (TIF) [file pone.0164592.s002.tif]

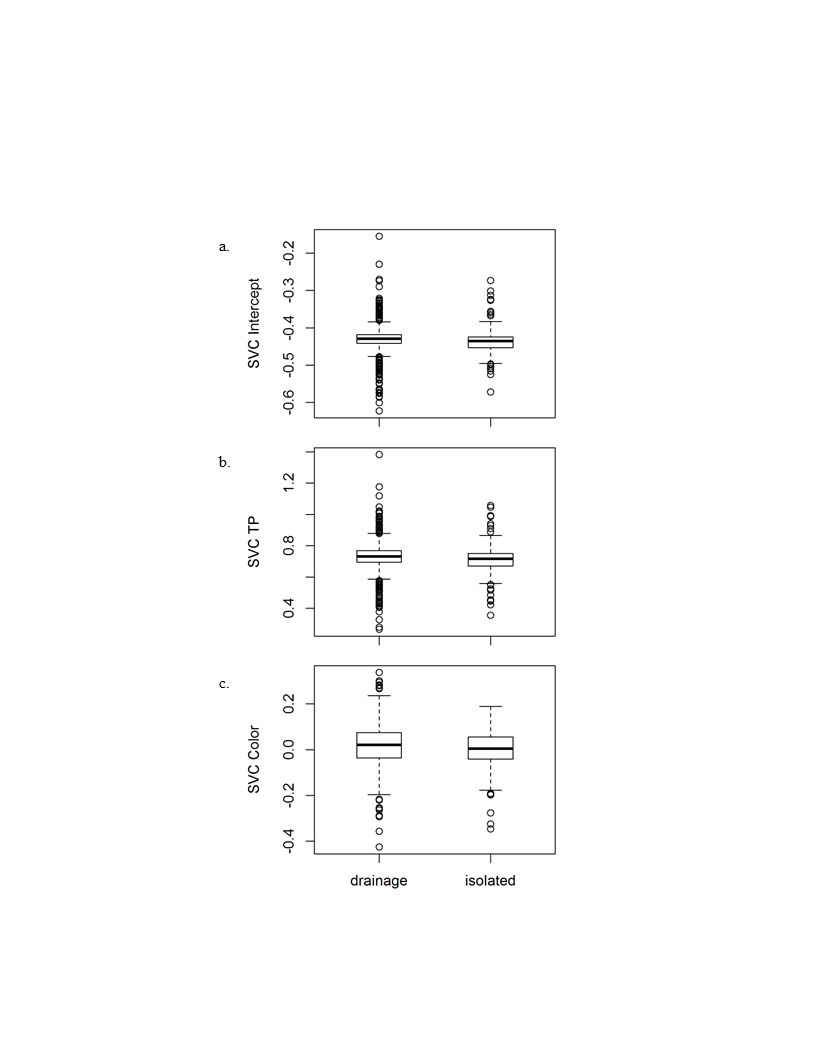

Supplement: S3 Fig — The distributions of spatially-varying coefficient values estimated in the SVCTP,COLOR model for a) spatially-varying intercept; b) TP; c) and water color coefficient values by lake connectivity type. Lakes are identified as drainage (i.e., presence of inflowing streams; N = 566 lakes) or isolated (i.e., no inflowing streams; N = 213 lakes). Mean values among lake connectivity types are significantly different from one another based on t-tests (α < 0.05). (TIF) [file pone.0164592.s003.tif]
