# Supplementary material for: Spatial Variation in Nutrient and Water Color Effects on Lake Chlorophyll at Macroscales
Source: PLoS One. 2016 Oct 13;11(10):e0164592. doi: 10.1371/journal.pone.0164592 (PMC5063324; doi:10.1371/journal.pone.0164592)
Supplement: S1 Table — Mean and standard deviation (sd) values for lake water chemistry and lake and catchment characteristics were quantified by lake connectivity type in the full dataset (N = 838 lakes). (DOCX) [file pone.0164592.s004.docx]

**S1 Table. Mean and standard deviation values by lake connectivity type.** Mean and standard deviation (sd) values for lake water chemistry and lake and catchment characteristics were quantified by lake connectivity type in the full dataset (N = 838 lakes).

| Variable | Isolated | Drainage |
| --- | --- | --- |
| Chlorophyll a (μg/L) | 12.29 (21.47) | 10.32 (18.60) |
| TP (μg/L) | 23.83 (27.20) | 21.49 (27.03) |
| Water color (PCU) | 20.40 (20.54) | 20.27 (21.17) |
|  |  |  |
| Max. depth (m) | 9.43 (8.00) | 12.07 (8.20) |
| Lake area (ha) | 43.93 (89.04) | 277.31 (637.60) |
| Catchment area (ha) | 256.91 (360.63) | 6175.76 (21555.39) |
| CA:LK | 8.51 (9.29) | 30.53 (126.44) |
|  |  |  |
| Prop. Agriculture | 0.05 (0.13) | 0.20 (0.19) |
| Prop. Urban | 0.14 (0.19) | 0.09 (0.14) |
| Prop. Wetland | 0.08 (0.10) | 0.11(0.11) |
| Prop. Forest | 0.65 (0.26) | 0.57 (0.22) |
