# Supplementary material for: Spatial Variation in Nutrient and Water Color Effects on Lake Chlorophyll at Macroscales
Source: PLoS One. 2016 Oct 13;11(10):e0164592. doi: 10.1371/journal.pone.0164592 (PMC5063324; doi:10.1371/journal.pone.0164592)
Supplement: S2 Table — Summary statistics on lake water chemistry variables and hypothesized lake and landscape covariates for lakes with significant positive water color–CHL relationships (N = 4 lakes) and significant negative relationships (N = 16 lakes). Prop. = proportion in the lake catchment. (DOCX) [file pone.0164592.s005.docx]

**S2 Table. Characteristics of lakes with positive and negative water color – CHL relationships.** Summary statistics on lake water chemistry variables and hypothesized lake and landscape covariates for lakes with significant positive water color–CHL relationships (N = 4 lakes) and significant negative relationships (N = 16 lakes). Prop. = proportion in the lake catchment.

| Variable | Mean | Median | Range | Standard deviation |
| --- | --- | --- | --- | --- |
| *Positive water color* |  |  |  |  |
| Chlorophyll a (μg/L) | 1.37 | 1.29 | 1.02 – 1.90 | 0.37 |
| TP (μg/L) | 4.63 | 4.71 | 2.10 – 7.00 | 2.04 |
| Water color (PCU) | 5.62 | 6.00 | 2.00 – 8.50 | 3.14 |
|  |  |  |  |  |
| Max. depth (m) | 27.81 | 26.82 | 11.89 – 45.72 | 14.90 |
| Lake area (ha) | 93.30 | 85.58 | 13.36 – 188.66 | 72.58 |
| Catchment area (ha) | 446.30 | 486.8 | 179.20 – 632.40 | 197.66 |
| CA:LK | 7.14 | 6.11 | 2.91 – 13.41 | 4.46 |
|  |  |  |  |  |
| Prop. Agriculture | 0 | 0 | 0 – 0 | 0 |
| Prop. Urban | 0.08 | 0.08 | 0 – 0.11 | 0.03 |
| Prop. Wetland | 0.01 | 0.02 | 0 – 0.02 | 0.01 |
| Prop. Forest | 0.71 | 0.72 | 0.58 – 0.80 | 0.01 |
|  |  |  |  |  |
| *Negative water color* |  |  |  |  |
| Chlorophyll a (μg/L) | 10.09 | 5.61 | 1.69 – 44.37 | 11.04 |
| TP (μg/L) | 18.65 | 13.12 | 6.62 – 42.75 | 12.28 |
| Water color (PCU) | 14.59 | 9.62 | 5.00 – 50.37 | 12.28 |
|  |  |  |  |  |
| Max. depth (m) | 10.56 | 9.40 | 4.00 – 23.30 | 5.49 |
| Lake area (ha) | 267.58 | 39.42 | 5.12 – 3179.20 | 780.71 |
| Catchment area (ha) | 8196.50 | 1081.20 | 61.30 – 104456.20 | 25765.36 |
| CA:LK | 54.82 | 19.09 | 3.93 – 434.84 | 112.08 |
|  |  |  |  |  |
| Prop. Agriculture | 0.21 | 0.21 | 0 – 0.45 | 0.15 |
| Prop. Urban | 0.08 | 0.05 | 0 – 0.46 | 0.11 |
| Prop. Wetland | 0.08 | 0.06 | 0 – 0.43 | 0.10 |
| Prop. Forest | 0.55 | 0.52 | 0.32 – 0.91 | 0.19 |
